# 2,2,2-Tribromo-*N*-phenyl­acetamide

**DOI:** 10.1107/S160053680903298X

**Published:** 2009-08-22

**Authors:** B. Thimme Gowda, Sabine Foro, P. A. Suchetan, Hartmut Fuess

**Affiliations:** a Department of Chemistry, Mangalore University, Mangalagangotri 574 199, Mangalore, India; b Institute of Materials Science, Darmstadt University of Technology, Petersenstrasse 23, D-64287 Darmstadt, Germany

## Abstract

In the title compound, C_8_H_6_Br_3_NO, the N—H bond is *anti* to the carbonyl bond in the side chain. The N—H hydrogen atom is involved in a two-centered bond as it shows simultaneous N—H⋯Br intra- and N—H⋯O inter­molecular inter­actions in the structure. In the crystal, mol­ecules are packed into column-like chains along the *b* axis through the N—H⋯O hydrogen bonds.

## Related literature

For the preparation of the compound, see: Gowda *et al.* (2003 ▶). For related structures, see: Brown *et al.* (1966 ▶); Dou *et al.* (1994 ▶); Gowda *et al.* (2007 ▶, 2009 ▶).
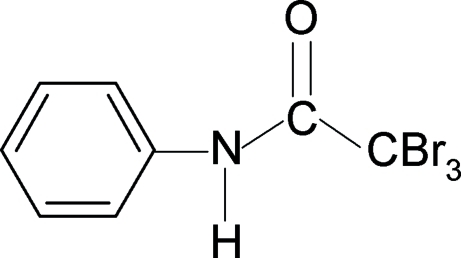


## Experimental

### 

#### Crystal data

C_8_H_6_Br_3_NO
*M* *_r_* = 371.87
Orthorhombic, 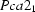

*a* = 10.1863 (8) Å
*b* = 9.1483 (7) Å
*c* = 11.8856 (9) Å
*V* = 1107.59 (15) Å^3^
*Z* = 4
Cu *K*α radiation
μ = 13.22 mm^−1^
*T* = 299 K
0.50 × 0.18 × 0.13 mm

#### Data collection

Enraf–Nonius CAD-4 diffractometer
Absorption correction: ψ scan (North *et al.*, 1968 ▶) *T*_min_ = 0.037, *T*_max_ = 0.178
2653 measured reflections
1311 independent reflections
1237 reflections with *I* > 2σ(*I*)
*R*_int_ = 0.052
3 standard reflections frequency: 120 min intensity decay: 1.5%

#### Refinement

*R*[*F* ^2^ > 2σ(*F* ^2^)] = 0.079
*wR*(*F* ^2^) = 0.237
*S* = 1.05
1311 reflections
119 parameters
25 restraints
H-atom parameters constrained
Δρ_max_ = 1.86 e Å^−3^
Δρ_min_ = −1.18 e Å^−3^
Absolute structure: Flack (1983 ▶), 276 Friedel pairs
Flack parameter: 0.00 (13)

### 

Data collection: *CAD-4-PC* (Enraf–Nonius, 1996 ▶); cell refinement: *CAD-4-PC*; data reduction: *REDU4* (Stoe & Cie, 1987 ▶); program(s) used to solve structure: *SHELXS97* (Sheldrick, 2008 ▶); program(s) used to refine structure: *SHELXL97* (Sheldrick, 2008 ▶); molecular graphics: *PLATON* (Spek, 2009 ▶); software used to prepare material for publication: *SHELXL97*.

## Supplementary Material

Crystal structure: contains datablocks I, global. DOI: 10.1107/S160053680903298X/fl2260sup1.cif

Structure factors: contains datablocks I. DOI: 10.1107/S160053680903298X/fl2260Isup2.hkl

Additional supplementary materials:  crystallographic information; 3D view; checkCIF report

## Figures and Tables

**Table 1:** Hydrogen-bond geometry (Å, °)

*D*—H⋯*A*	*D*—H	H⋯*A*	*D*⋯*A*	*D*—H⋯*A*
N1—H1*N*⋯O1^i^	0.86	2.19	2.967 (13)	150
N1—H1*N*⋯Br1	0.86	2.68	3.123 (13)	114

Symmetry code: (i) 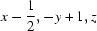.

**Fig. 1.**
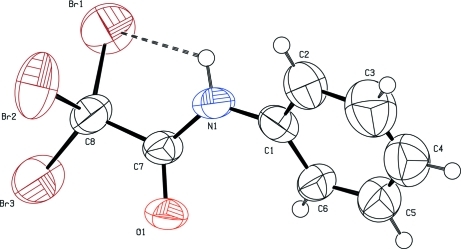
Molecular structure of (I), showing the atom labelling scheme. Displacement ellipsoids are drawn at the 50% probability level and H atoms are represented as small spheres of arbitrary radii.

**Fig. 2.**
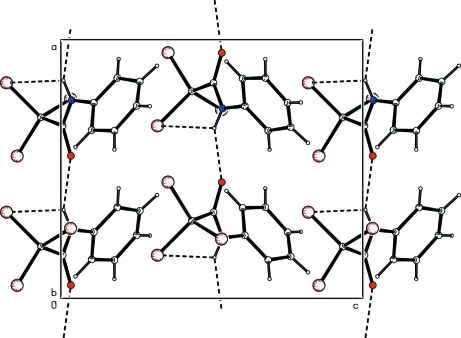
Molecular packing of (I) with hydrogen bonds shown as dashed lines.

## supplementary crystallographic information

### Comment

The structure of (I) has been determined (Fig. 1) as part of a study on the effect of ring and side chain substituents on the structures of *N*-aromatic amides (Dou *et al.*, 1994; Gowda *et al.*, 2007, 2009). The   N—H bond in (I) is *anti* to the C=O bond in the side chain, similar to that observed in *N*-(phenyl)acetamide (Brown, 1966), 2,2,2-trichloro-*N*-(phenyl)acetamide (Dou *et al.*, 1994), 2,2,2-trimethyl-*N*-(phenyl)acetamide (Gowda *et al.*, 2007) and other amides (Gowda *et al.*, 2009). The N—H hydrogen atom is involved as the donor in a  two-centered bond; an intramolecular N—H···Br bond and an intermolecular N—H···O bond (Table 1).  The  N1—H1N···O1 bonds involved in the formation of molecular chains in the direction of the *b*-axis are shown Fig. 2.

### Experimental

The title compound was prepared from aniline, tribromoacetic acid and phosphorylchloride according to the literature method (Gowda *et al.*, 2003). The purity of the compound was checked by determining its melting point. It was further characterized by recording its infrared spectra. Single crystals of the title compound used for X-ray diffraction studies were obtained by a slow evaporation from petroleum ether at room temperature.

### Refinement

The H atoms were positioned with idealized geometry using a riding model [N—H = 0.86 Å, C—H = 0.93 Å]. All H atoms were refined with isotropic displacement parameters (set to 1.2 times of the *U*_eq_ of the parent atom).

The U^ij^ components of C2, C3, C4 and C5 were restrained to approximate isotropic behavoir.

The residual electron-density features are located in the region of Br3 and Br1. The highest peak is 1.25 Å from Br3 and the deepest hole is 0.75 Å from Br1.

### Crystal data

**Table:**

C_8_H_6_Br_3_NO	*F*(000) = 696
*M**_r_* = 371.87	*D*_x_ = 2.230 Mg m^−^^3^
Orthorhombic, *P**c**a*2_1_	Cu *K*α radiation, λ = 1.54180 Å
Hall symbol: P 2c -2ac	Cell parameters from 25 reflections
*a* = 10.1863 (8) Å	θ = 4.8–20.7°
*b* = 9.1483 (7) Å	µ = 13.22 mm^−^^1^
*c* = 11.8856 (9) Å	*T* = 299 K
*V* = 1107.59 (15) Å^3^	Needle, colourless
*Z* = 4	0.50 × 0.18 × 0.13 mm

### Data collection

**Table:**

Enraf–Nonius CAD-4 diffractometer	1237 reflections with *I* > 2σ(*I*)
Radiation source: fine-focus sealed tube	*R*_int_ = 0.052
graphite	θ_max_ = 67.0°, θ_min_ = 4.8°
ω/2θ scans	*h* = −12→0
Absorption correction: ψ scan (North *et al.*, 1968)	*k* = −10→10
*T*_min_ = 0.037, *T*_max_ = 0.178	*l* = −11→14
2653 measured reflections	3 standard reflections every 120 min
1311 independent reflections	intensity decay: 1.5%

### Refinement

**Table:**

Refinement on *F*^2^	Hydrogen site location: inferred from neighbouring sites
Least-squares matrix: full	H-atom parameters constrained
*R*[*F*^2^ > 2σ(*F*^2^)] = 0.079	*w* = 1/[σ^2^(*F*_o_^2^) + (0.1659*P*)^2^ + 2.9656*P*] where *P* = (*F*_o_^2^ + 2*F*_c_^2^)/3
*wR*(*F*^2^) = 0.237	(Δ/σ)_max_ = 0.002
*S* = 1.05	Δρ_max_ = 1.86 e Å^−^^3^
1311 reflections	Δρ_min_ = −1.18 e Å^−^^3^
119 parameters	Extinction correction: *SHELXL97* (Sheldrick, 2008), Fc^*^=kFc[1+0.001xFc^2^λ^3^/sin(2θ)]^-1/4^
25 restraints	Extinction coefficient: 0.0035 (8)
Primary atom site location: structure-invariant direct methods	Absolute structure: Flack (1983), 276 Friedel pairs
Secondary atom site location: difference Fourier map	Flack parameter: 0.00 (13)

### Special details

**Table:**

Geometry. All e.s.d.'s (except the e.s.d. in the dihedral angle between two l.s. planes) are estimated using the full covariance matrix. The cell e.s.d.'s are taken into account individually in the estimation of e.s.d.'s in distances, angles and torsion angles; correlations between e.s.d.'s in cell parameters are only used when they are defined by crystal symmetry. An approximate (isotropic) treatment of cell e.s.d.'s is used for estimating e.s.d.'s involving l.s. planes.
Refinement. Refinement of *F*^2^ against ALL reflections. The weighted *R*-factor *wR* and goodness of fit *S* are based on *F*^2^, conventional *R*-factors *R* are based on *F*, with *F* set to zero for negative *F*^2^. The threshold expression of *F*^2^ > σ(*F*^2^) is used only for calculating *R*-factors(gt) *etc*. and is not relevant to the choice of reflections for refinement. *R*-factors based on *F*^2^ are statistically about twice as large as those based on *F*, and *R*- factors based on ALL data will be even larger.

### Fractional atomic coordinates and isotropic or equivalent isotropic displacement parameters (Å^2^)

**Table:**

	*x*	*y*	*z*	*U*_iso_*/*U*_eq_	
C1	0.2439 (16)	0.3139 (13)	0.6091 (14)	0.057 (3)	
C2	0.1483 (19)	0.2891 (18)	0.6869 (19)	0.078 (4)	
H2	0.0753	0.3500	0.6914	0.094*	
C3	0.162 (3)	0.169 (3)	0.761 (3)	0.110 (7)	
H3	0.1010	0.1557	0.8185	0.132*	
C4	0.256 (3)	0.077 (2)	0.751 (2)	0.095 (5)	
H4	0.2575	−0.0070	0.7947	0.114*	
C5	0.3521 (19)	0.1043 (17)	0.6774 (19)	0.079 (4)	
H5	0.4247	0.0427	0.6773	0.095*	
C6	0.3495 (16)	0.2176 (13)	0.6019 (17)	0.065 (4)	
H6	0.4153	0.2297	0.5484	0.078*	
C7	0.3328 (12)	0.5181 (14)	0.5056 (12)	0.053 (3)	
C8	0.3002 (15)	0.6556 (17)	0.4339 (16)	0.069 (4)	
N1	0.2341 (10)	0.4331 (11)	0.5359 (12)	0.058 (2)	
H1N	0.1579	0.4526	0.5085	0.069*	
O1	0.4489 (8)	0.5008 (11)	0.5332 (12)	0.076 (4)	
Br1	0.1668 (3)	0.6121 (3)	0.3197 (2)	0.0988 (9)	
Br2	0.2294 (3)	0.80125 (17)	0.5320 (2)	0.1007 (10)	
Br3	0.4497 (2)	0.7263 (3)	0.3550 (3)	0.1230 (14)	

### Atomic displacement parameters (Å^2^)

**Table:**

	*U*^11^	*U*^22^	*U*^33^	*U*^12^	*U*^13^	*U*^23^
C1	0.049 (6)	0.064 (7)	0.059 (8)	−0.012 (6)	−0.003 (6)	0.003 (6)
C2	0.075 (8)	0.080 (7)	0.079 (8)	−0.011 (6)	0.014 (7)	0.021 (6)
C3	0.112 (11)	0.110 (9)	0.108 (11)	−0.016 (8)	0.014 (9)	0.020 (8)
C4	0.106 (9)	0.085 (7)	0.095 (10)	−0.013 (8)	−0.013 (8)	0.014 (7)
C5	0.082 (8)	0.069 (6)	0.087 (9)	−0.002 (6)	−0.016 (7)	0.004 (6)
C6	0.060 (8)	0.052 (6)	0.083 (10)	0.004 (5)	−0.022 (7)	0.001 (6)
C7	0.038 (5)	0.066 (6)	0.054 (7)	0.001 (4)	0.005 (5)	0.017 (6)
C8	0.057 (8)	0.068 (7)	0.081 (11)	0.014 (6)	0.006 (7)	0.020 (7)
N1	0.037 (5)	0.068 (5)	0.068 (7)	0.005 (4)	−0.007 (5)	0.008 (6)
O1	0.035 (4)	0.085 (6)	0.109 (10)	−0.008 (4)	−0.007 (5)	0.042 (7)
Br1	0.1101 (17)	0.1157 (14)	0.0705 (12)	−0.0051 (11)	−0.0328 (12)	0.0179 (10)
Br2	0.164 (2)	0.0664 (9)	0.0719 (12)	0.0155 (10)	0.0171 (14)	0.0000 (8)
Br3	0.0680 (11)	0.1321 (19)	0.169 (3)	0.0095 (10)	0.0356 (14)	0.091 (2)

### Geometric parameters (Å, °)

**Table:**

C1—C2	1.36 (3)	C5—H5	0.9300
C1—C6	1.39 (2)	C6—H6	0.9300
C1—N1	1.399 (19)	C7—O1	1.237 (16)
C2—C3	1.42 (3)	C7—N1	1.321 (16)
C2—H2	0.9300	C7—C8	1.555 (18)
C3—C4	1.27 (4)	C8—Br3	1.902 (16)
C3—H3	0.9300	C8—Br2	1.913 (17)
C4—C5	1.34 (4)	C8—Br1	1.960 (19)
C4—H4	0.9300	N1—H1N	0.8600
C5—C6	1.37 (2)		
			
C2—C1—C6	119.3 (15)	C5—C6—C1	116.9 (18)
C2—C1—N1	120.1 (15)	C5—C6—H6	121.6
C6—C1—N1	120.6 (15)	C1—C6—H6	121.6
C1—C2—C3	118.7 (19)	O1—C7—N1	125.5 (11)
C1—C2—H2	120.6	O1—C7—C8	117.0 (11)
C3—C2—H2	120.6	N1—C7—C8	117.5 (11)
C4—C3—C2	122 (3)	C7—C8—Br3	111.9 (9)
C4—C3—H3	119.2	C7—C8—Br2	108.1 (11)
C2—C3—H3	119.2	Br3—C8—Br2	111.4 (9)
C3—C4—C5	119 (2)	C7—C8—Br1	111.3 (11)
C3—C4—H4	120.3	Br3—C8—Br1	106.4 (9)
C5—C4—H4	120.3	Br2—C8—Br1	107.6 (7)
C4—C5—C6	123.6 (18)	C7—N1—C1	125.0 (11)
C4—C5—H5	118.2	C7—N1—H1N	117.5
C6—C5—H5	118.2	C1—N1—H1N	117.5
			
C6—C1—C2—C3	2(3)	N1—C7—C8—Br3	−159.7 (12)
N1—C1—C2—C3	−179.1 (19)	O1—C7—C8—Br2	−100.0 (14)
C1—C2—C3—C4	−5(4)	N1—C7—C8—Br2	77.2 (16)
C2—C3—C4—C5	8(4)	O1—C7—C8—Br1	142.1 (13)
C3—C4—C5—C6	−7(4)	N1—C7—C8—Br1	−40.7 (17)
C4—C5—C6—C1	4(3)	O1—C7—N1—C1	4(3)
C2—C1—C6—C5	−2(2)	C8—C7—N1—C1	−173.4 (14)
N1—C1—C6—C5	179.6 (15)	C2—C1—N1—C7	139.6 (18)
O1—C7—C8—Br3	23.1 (19)	C6—C1—N1—C7	−42 (2)

### Hydrogen-bond geometry (Å, °)

**Table:**

*D*—H···*A*	*D*—H	H···*A*	*D*···*A*	*D*—H···*A*
N1—H1N···O1^i^	0.86	2.19	2.967 (13)	150
N1—H1N···Br1	0.86	2.68	3.123 (13)	114

Symmetry codes: (i) *x*−1/2, −*y*+1, *z*.
